# Duration of onset, body temperature and C-reactive protein can be used to predict the results of pus culture in children with acute osteomyelitis of long bones

**DOI:** 10.1186/s13052-024-01804-9

**Published:** 2024-11-05

**Authors:** Haiting Jia, Yanan Liu, Tao Liu

**Affiliations:** 1 Department of Orthopaedic Trauma Surgery, Children’s Hospital Affiliated to Shandong University (Jinan Children’s Hospital), Jinan, Shandong 250022 China; 2 Department of Orthopaedic Surgery, Weifang Yidu Central Hospital, Weifang, Shandong 262500 China

**Keywords:** Osteomyelitis, Culture-negative, Pediatric

## Abstract

**Background:**

With the application of PCR testing and Metagenomic Next-Generation Sequencing(mNGS), the detection rate of causative organisms in paediatric bone and joint infections has been greatly improved. The aim of our study is to identify some indicators that could be used to distinguish the culture results to optimize the use of PCR and mNGS.

**Methods:**

In this study, a total of 117 cases of acute osteomyelitis of long bones in children who underwent pus culture were included. Patients were grouped as culture-negative (n:21) and culture-positive (n:96) groups according to the results of pus culture. Age, sex, duration of onset, maximum body temperature at onset, inflammatory indicators and D-dimer after admission were systematically collected for all patients and were compared for both groups. ROC curve (ROC) was used to evaluate the diagnostic efficiency of culture-negative. Logistic regression analysis was conducted to determine independent risk factors for culture-negative.

**Results:**

There was no significant difference in age, sex and erythrocyte sedimentation rate between culture-negative group and culture-positive group (*P* > 0.05). The duration of onset was longer, and the temperature, white blood cells, neutrophils count, C-reactive protein and D-dimer were less elevated in culture-negative acute osteomyelitis (*P* < 0.05). Duration of onset, maximum body temperature at onset, white blood cell count, neutrophil count, C-reactive protein, and D-dimer have certain diagnostic efficacy in judging the efficacy of negative culture. Logistic regression analysis indicated that the duration of onset more than 6.5 days, the maximum body temperature at onset lower than 38.35℃ and C-reactive protein lower than 78.40 mg/L were independent risk factors for negative culture (*P* < 0.05).

**Conclusions:**

Our study revealed that duration of onset more than 6.5 days, maximum body temperature at onset lower than 38.35℃ and C-reactive protein lower than 78.40 mg/L were independent risk factors for predicting negative culture. In children with this type of acute osteomyelitis, we recommend that the pus be tested by PCR or mNGS as a priority.

## Background

Pediatric osteomyelitis is a common infectious disease in children, with an incidence between 1:10000 and 1:5000, mainly in boys [1, 2]. According to the time of onset, within 2 weeks is acute, 2 weeks to 3 months is subacute, and more than 3 months is chronic [3]. The clinical manifestations of osteomyelitis are redness, swelling, increased skin temperature, and limited limb activity. If the treatment is not timely or improper, it can cause pathological fracture and limb deformity [4].

For osteomyelitis, identification of a causative microorganism is essential to diagnosis and determine the appropriate antibiotic therapy. Causative bacterial pathogens are identified primarily through bacterial cultures of blood and infected musculoskeletal sources. Blood cultures identify a pathogen in < 50% of patients. Concurrent collection of source samples from infected tissues increases the chance of identifying a causative bacterial pathoge [5]. Staphylococcus aureus is the most common pathogenic microorganism of osteomyelitis, accounting for 70–90% of culture-positive cases. However, despite attempts to identify the pathogen, no pathogenic microorganism was found in 24–68% of osteomyelitis [6].

To improve the detection rate of pathogenic microorganisms, PCR-based testing and Metagenomic next-generation sequencing (mNGS) are used, especially in culture-negative cases. Ramchandar et al. [7]conducted mNGS, PCR and routine culture on samples of 42 children with bone and joint infection. Pathogenic microorganisms were detected in 61.9% of the children through mNGS, while only 45.2% identified pathogens through routine culture. *Kingella kingae* was detected by mNGS and PCR in 5 children with negative culture. Although PCR and mNGS have greatly improved the detection rate of pathogenic microorganisms, there are still some cases where pathogenic microorganisms have not been found. Recent 2021 AHO clinical practice guidelines from the Pediatric Infectious Disease Society mentions that these tests remain adjunctive to and not replacements for standard cultures [8].With economic considerations, we tried to identify certain indicators that could be used to predict culture negative and positive, and to optimize the use of PCR and mNGS by performing PCR or mNGS tests for potential culture negative cases.

## Materials and methods

### Patients

The data of 117 cases of acute osteomyelitis in children diagnosed and treated from July 2017 to March 2024 were retrospectively analyzed. According to the results of pus culture, 21 cases were negative and 96 cases were positive. Inclusion criteria: (1) Duration of onset within 2 weeks; (2) The affected sites were all in the long bones of the limbs; (3) Standard culture and placement of a portion of specimens into aerobic blood culture bottles were performed on the pus; (4) Complete clinical data. Exclusion criteria: (1) duration of onset more than 2 weeks; (2) Diseases of the immune system and blood system, bone tumors, open fractures, and osteomyelitis caused by mycobacteria or fungi.

### Pus collection

As indicated in the inclusion criteria, pus was collected in all 117 patients. After completing blood culture and blood index examination, all children were given empirical antibiotic therapy. Then, the invasive diagnostic procedures (including ultrasound-guided puncture and surgery) were performed to obtain pus based on the imaging results. The obtained pus was immediately sent to the department of microbiology and standard culture (Constant temperature culture at 35℃) and placement of a portion of specimens into aerobic blood culture bottles (by creating the conditions for the growth and reproduction of microorganisms, the detection rate of pathogenic microorganisms can be improved) were performed on the pus. Results of pus culture and drug sensitivity will be available within 2–5 days.

### Risk factors and definition of terms

Clinical data of children in the culture-negative group and culture-positive group were collected and retrospectively analyzed. Maximum body temperature (from onset to admission), white blood cell count (WBC), neutrophil count, C-reactive protein (CRP), erythrocyte sedimentation rate (ESR) and D-dimer detected after admission, age, gender, duration of onset before admission were compared between the two groups.

### Statistical analysis

SPSS22.0 statistical software was used for data analysis. Measurement data conforming to normal distribution were expressed as mean ± standard deviation, and two independent samples T-test was used for comparison. For the non-normal distribution measurement data, the median and quartile spacing were expressed, and the rank sum test was used for inter-group comparison. Counting data were expressed as percentage/rate, and the X^2^ test was used for comparison. Receiver operator curves (ROC) were constructed to identify cut points with optimal sensitivity and specificity for the variables found to remain significant in multivariate analysis. Sensitivity and specificity were calculated based on the suggested ROC cut points. Logistic regression analysis was used to determine the independent risk factors for negative culture. A p value < 0.05 was considered statistically significant.

## Results

### General information

In the culture-negative group, there were 15 boys and 6 girls, aged from 4 months to 11 years old, 20 cases had fever before admission, 9 cases of femur, 8 cases of tibia, 2 cases of humerus, 1 case of fibula and 1 case of radius. There were 13 cases with elevated WBC, 12 cases with elevated neutrophil count, 19 cases with elevated CRP, 20 cases with elevated ESR and 12 cases with elevated D-dimer. In the culture-positive group, there were 57 boys and 39 girls, aged from 19 days to 15 years old, 96 cases had fever before admission, 42 cases of tibia, 38 cases of femur, 7 cases of humerus, 5 cases of fibula, 3 cases of radius and 1 case of ulna. There were 77 cases with elevated WBC, 76 cases with elevated neutrophil count, 92 cases with elevated CRP, 95 cases with elevated ESR and 69 cases with elevated D-dimer. The pathogenic microorganisms were methicillin-sensitive *Staphylococcus aureus* in 63 cases, methicillin-resistant Staphylococcus aureus in 29 cases, *Streptococcus* pneumoniae in 2 cases and *Salmonella* enteritidis in 2 cases.

### Comparative analysis results

There was no significant difference in age, sex and ESR between culture-negative group and culture-positive group (*P* > 0.05). The median duration of onset in culture-negative group and culture-positive group was 7 and 5 days, the median maximum body temperature at onset was 38.0℃ and 39.5℃, the median WBC was 11.79 × 10^9^/L and 14.77 × 10^9^/L, the median neutrophil count was 7.69 × 10^9^/L and 9.81 × 10^9^/L, the median CRP was 45.39 mg/L and 67.43 mg/L, and the median D-dimer was 1.12 mg/L and 1.50 mg/L, respectively, with statistical significance (*P* < 0.05). Table 1.

**Table 1:** Observation indicators and statistical analysis results

	Culture negative (*n* = 21)	Culture positive (*n* = 96)	z/x2	*P*
Age(m)	72.00(24.50,120.00)	49.50(19.25,94.25)	-1.230	0.219
Sex				
Male	15	57	1.058	0.304
Female	6	39		
Duration of symptoms(d)	7.00(5.00,10.50)	5.00(4.00,7.75)	-2.219	0.026
Maximum body temperature(℃)	38.00(38.80,39.75)	39.50(38.73,40.00)	-2.180	0.029
WBC(×10^9^/L)	11.79(7.87,13.81)	14.77(11.18,18.05)	-2.237	0.025
Neutrophil (×10^9^/L)	7.69(4.29,9.63)	9.81(6.90,13.66)	-2.397	0.017
CRP(mg/L)	45.39(16.66,71.15)	67.43(35.34,112.62)	-2.319	0.020
ESR(mm/h)	56.00(40.50,76.50)	60.00(43.00,77.00)	−0.583	0.560
D- dimer(mg/L)	1.12(0.64,2.01)	1.50(0.88,3.80)	−1.992	0.046

### ROC curve results

ROC curves of duration of onset, maximum body temperature at onset, WBC, neutrophils count, CRP and D-dimer to predict negative culture were all of certain diagnostic efficacy (*P* < 0.05). The optimal cutoff of the duration of onset was 6.5 days, and the sensitivity, specificity and area under the curve were 38.1%, 90.6% and 0.654 (0.512 to 0.796), respectively. The optimal cutoff of the maximum body temperature at onset was 38.35℃, and the sensitivity, specificity and area under the curve were 41.4%, 90.3% and 0.652 (0.510–0.793), respectively. The optimal cutoff of WBC was 13.25 × 10^9^/L, and the sensitivity, specificity and area under the curve were 76.2%, 62.5% and 0.656 (0.524–0.788), respectively. The optimal cutoff of neutrophil count was 9.73 × 10^9^/L, and the sensitivity, specificity and area under the curve were 81.0%, 51.0% and 0.667 (0.531–0.803), respectively. The optimal cutoff of CRP was 78.40 mg/L, and the sensitivity, specificity and area under the curve were 90.5%, 42.7% and 0.662 (0.537–0.787), respectively. The optimal cutoff of D-dimer was 2.29 mg/L, and the sensitivity, specificity and area under the curve were 90.5%, 39.6% and 0.639 (0.522–0.756), respectively. The sensitivity, specificity and area under the curve were 90.5%, 65.6% and 0.822 (0.723–0.921), respectively, when the six indexes were combined.

### Logistic regression analysis results

The optimal cutoff of the six indexes were divided into binary variables for logistic regression analysis, which indicated that the duration of onset more than 6.5 days (OR = 9.215, 95%CI: 1.927–44.073), the maximum body temperature at onset lower than 38.35℃ (OR = 10.547, 95%CI: 2.057–54.070) and CRP lower than 78.40 mg/L (OR = 8.000, 95%CI: 1.502–42.602) were independent risk factors for negative culture (*P* < 0.05). Table 2.

**Table 2:** Logistic regression analysis in predicting negative culture

	β	SE	Wald	95% Exp(B)	*P*
Duration of onset>6.5 days	2.221	0.799	7.735	9.215(1.927–44.073)	0.005
Maximum body temperature at onset <38.35 ℃	2.356	0.834	7.982	10.547(2.057–54.070)	0.005
WBC<13.25 × 10^9^/L	2.022	1.172	2.974	7.552(0.759–75.160)	0.085
Neutrophil count<9.73 × 10^9^/L	−0.075	1.272	0.003	0.928(0.077–11.233)	0.953
CRP<78.40 mg/L	2.079	0.853	5.938	8.000(1.502–42.602)	0.015
D-dimer<2.29 mg/L	1.597	0.873	3.346	4.937(0.892–27.325)	0.067

## Discussion

The incidence of acute osteomyelitis in children varies between 1:5000 and 1:10,000, with boys having a rate twice that of girls [1]. Pediatric osteomyelitis tends to occur in the metaphysis of the long bones, with the femur and tibia being the most common sites. In our study, boys had a higher proportion (72/117), and tibia and femur were the most common sites (97/117).

The Pediatric Infectious Disease Society (PIDS) proposed updated guidelines in 2021 for the management of acute osteomyelitis, recommending blood and tissue cultures for all acute osteomyelitis cases [8]. However, because the positive rate of blood culture is low, the guidelines recommend invasive diagnosis to obtain samples such as pus and bone tissue for bacterial culture to increase the positive rate. In our study, we performed blood and pus culture (including standard culture and placement of a portion of specimens into aerobic blood culture bottles) in 117 children with acute osteomyelitis, with an overall positive rate of 82.1% (96/117). Despite these efforts, we still fail to find the bacterial culprit about in 1 in 5 children. Because blood cultures of hand and foot osteomyelitis are often negative, and it is difficult to obtain cultures with less pus, we only selected osteomyelitis of the long bones of the limbs in our study. It has been suggested that yield of Kingella kingae cultures can be improved by inoculating specimens into aerobic blood culture vials [9], and in our study the 117 patients with acute osteomyelitis followed this recommendation. However, despite attempts to culture we did not find *Kingella kingae*. Searching the previous published literature in our country, no relevant reports on osteomyelitis of Kingella kingae were found, considering that the bacteria may be related to regional distribution. Due to economic and technical limitations, PCR and mNGS were not used in our study.

WBC, ESR and CRP are commonly used indicators of bacterial infection [10]. The role of procalcitonin in bone and joint infections is unclear and is not recommended [8]. In the culture-negative group, 61.9% (13/21) showed elevated WBC, 57.1% (12/21) showed elevated neutrophil count, 90.5% (19/21) showed elevated CRP, and 95.2% (20/21) showed elevated ESR. In the culture-positive group, 80.2%(77/96) showed elevated WBC, 79.2% (76/96) showed elevated neutrophil count, 95.8% (92/96) showed elevated CRP, and 99.0% (95/96) showed elevated ESR. Except for neutrophil counts, there were no statistically significant differences between the two groups in the proportion of elevated WBC, CRP, and ESR. Due to the low sensitivity of ESR and CRP in smaller bone infections like calcaneal osteomyelitis [11], in order to increase the comparability, the patients we selected for study were all affected in the long bones of the limbs. Therefore, the proportion of elevated inflammatory markers in our study were higher than previously reported in the literature.

D-dimer is the product of fibrin degradation, and the increase of D-dimer indicates that the body is in a state of hypercoagulation and hyperfibrinolysis. In our study, D-dimer was increased in 57.1% (12/21) of culture-negative group and 71.9%(69/96) of culture-positive group. Although D-dimer positivity did not differ between the two groups, the proportion of D-dimer elevation was higher in the culture-positive group than in the culture-negative group. Further comparative analysis showed that the level of D-dimer in the culture-positive group was significantly higher than that in the culture-negative group. The reason was that most of the pathogenic microorganisms in the culture-positive group were *Staphylococcus aureus*. *Staphylococcus aureus* activates prothrombin by secreting staphylocoagulase and von willebrand factor binding protein, catalyzes the conversion of fibrinogen to fibrin, and also secretes staphylokinase to activate fibrinolytic system [12–15].

In our study, through comparative analysis we found that those in the culture-negative group admitted to hospital relatively later and had lower levels of fever, WBC, neutrophils count, CRP and D-dimer. It indicates that the culture-negative osteomyelitis is relatively mild. By drawing the ROC curve, we found that duration of onset, maximum body temperature at onset, WBC, neutrophils count, CRP and D-dimer had certain diagnostic performance, and the combined diagnostic efficiency of these 6 indicators was higher. We further performed logistic regression analysis and found that the duration of onset more than 6.5 days, the maximum body temperature at onset lower than 38.35℃, and CRP lower than 78.40 mg/L were independent risk factors for negative culture. The odds ratio for duration of onset cutoff in distinguishing between culture-negative and culture-positive was 9.215 (95% CI 1.927–44.073). The odds ratio for maximum body temperature at onset cutoff in distinguishing between culture-negative and culture-positive was 10.547 (95% CI 2.057–54.070). The odds ratio for CRP cutoff in distinguishing between culture-negative and culture-positive was 8.000 (95% CI 1.502–42.602).

For the choice of antibiotics for culture-negative osteomyelitis, Searns et al. [5]empirically treated most culture-negative osteomyelitis (91%) with cephalexin, and the readmission rate was similar to that of culture-positive osteomyelitis, suggesting that the treatment of culture-negative osteomyelitis is similar to that of osteomyelitis with identified pathogenic microorganism. Floyed et al. [16] recommend a short course of intravenous cefazolin for cultural-negative osteomyelitis followed by oral cephalexin. We selected cefazolin as the antibiotic for children with culture-negative osteomyelitis and their outcomes were good.

Our study had several limitations. First, PCR testing and mNGS were not used in culture-negative cases, which may cause potential selection bias. Second, the selected indicators in our study were examined after admission, and the disease course was different, so the selected indicators may be in different stages of the disease. Third, the use of antibiotics may affect the results of pus culture. It has been reported in the literature that antibiotic administration before surgery does not decrease surgical culture yield [17]. There are also literature reports that there was no difference in bone biopsy cultures for children who did and did not receive antibiotics before biopsy, but longer antibiotic durations before biopsy were associated with reduced culture yield [18].

## Conclusion

Compared with the culture-positive acute osteomyelitis, the duration of onset was longer, and the temperature, WBC, neutrophils count, CRP and D-dimer were less elevated in culture-negative acute osteomyelitis. Duration of onset more than 6.5 days, maximum body temperature at onset lower than 38.35℃ and C-reactive protein lower than 78.40 mg/L were independent risk factors for predicting negative culture. The results of our study can be used to guide the empirical administration of negative osteomyelitis on the one hand, and optimize the use of PCR testing and mNGS on the other hand.

## Data Availability

The datasets used and/or analyzed during the current study are available from the corresponding author on reasonable request.

## Abbreviations

CRP: C-reactive protein
ESR: Erythrocyte sedimentation rate
mNGS: Metagenomic Next-Generation Sequencing
WBC: White blood cell
